# Pertinence of glioma and single nucleotide polymorphism of TERT, CCDC26, CDKN2A/B and RTEL1 genes in glioma: a meta-analysis

**DOI:** 10.3389/fonc.2023.1180099

**Published:** 2023-09-07

**Authors:** Yaqi Wu, Jun Zhou, Jun Zhang, Zhijian Tang, Xi Chen, Lulu Huang, Shengwen Liu, Hong Chen, Yu Wang

**Affiliations:** ^1^ Department of Neurosurgery, Tongji Hospital, Tongji Medical College, Huazhong University of Science and Technology, Wuhan, China; ^2^ School of Health, Brooks College, Sunnyvale, CA, United States; ^3^ Department of Epidemiology and Statistics, School of Public Health, Medical College, Zhejiang University, Hangzhou, China; ^4^ Medical Affairs, the Department of ICON Pharma Development Solutions (IPD), ICON Public Limited Company (ICON Plc), Beijing, China; ^5^ Dediatric Department, School of Clinical Medicine for Women and Children, China Three Gorges University, Yichang Maternal and Child Health Hospital, Yichang, China

**Keywords:** glioma, single nucleotide polymorphism, risk, meta-analysis, genetic model

## Abstract

**Background:**

Previous genetic-epidemiological studies considered *TERT* (rs2736100), *CCDC26* (rs4295627), *CDKN2A/B* (rs4977756) and *RTEL1* (rs6010620) gene polymorphisms as the risk factors specific to glioma. However, the data samples of previous genetic-epidemiological studies are modest to determine whether they have definite association with glioma.

**Method:**

The study paid attention to systematically searching databases of PubMed, Embase, Web of Science (WoS), Scopus, Cochrane Library and Google Scholars. Meta-analysis under 5 genetic models, namely recessive model (RM), over-dominant model (O-DM), allele model (AM), co-dominant model (C-DM) and dominant model (DM) was conducted for generating odds ratios (ORs) and 95% confidence intervals (CIs). That was accompanied by subgroup analyses according to various racial groups. The software STATA 17.0 MP was implemented in the study.

**Result:**

21 articles were collected. According to data analysis results, in four genetic models (AM, RM, DM and C-DM) *TERT* gene rs2736100 polymorphism, *CCDC26* gene rs4295627 polymorphism, *CDKN2A/B* gene rs4977756 polymorphism and *RTEL1* gene rs6010620 polymorphisms increased the risk of glioma in Caucasians to different degrees. In Asian populations, the *CCDC26* gene rs4295627 polymorphism and *CDKN2A/B* gene rs4977756 polymorphism did not exhibit a relevance to the risk of glioma. It is suggested to cautiously explain these results as the sample size is small.

**Conclusion:**

The current meta-analysis suggested that the SNP of *TERT* (rs2736100), *CCDC26* (rs4295627), *CDKN2A/B* (rs4977756) and *RTEL1* (rs6010620) genes in glioma might increase risk of glioma, but there are ethnic differences. Further studies evaluating these polymorphisms and glioma risk are warranted.

## 1 Introduction

Gliomas, which occupy more than 80% primary malignant tumors of central nervous system, are presumably caused by the aberrant growth of glial cells, mostly in the brain and spinal cord (1). Based on the cell type from which they originate, gliomas may be divided into several categories such astrocytomas, oligodendrogliomas, and ependymomas. The World Health Organization first used molecular pathology in the diagnostic classification of gliomas in 2016(2). Adult diffuse glioma currently has 3 molecular subtypes, according to the most recent 2021 version: glioblastoma multiforme (GBM), isocitrate dehydrogenase-wild-type (IDH-W-T); astrocytoma, IDH-mutant; and oligodendroglioma, IDH-mutant and 1p/19q-codeleted (3). Major current treatment of glioma is the combination of surgery with radiotherapy and chemotherapy, and for certain patients, immunotherapy and targeted therapy were implemented additionally (4–8). Nevertheless, the median survival time for gliomas is approximate 12 to 15 months merely, and both mortality and morbidity are tremendous (9).

Glioma occurs due to both environmental factors and genetic factors, and its precise pathogenesis remains opaque (10, 11). Risk factors for glioma may include smoking, nitrosamines, race, ionizing radiation, and brain injuries (12). Single nucleotide polymorphisms (SNPs) in genetic variables partially increase glioma risk to some extent (10). Genome-related studies have materialized some advancements in the identification of the risk of glioma with the development of genomic research, which reveal the link between numerous SNPs and glioma risk, including 5p15.33 (rs2736100, *TERT*), 8q24.21 (rs4295627, *CCDC26*), 9p21.3 (rs4977756, *CDKN2A/B*), and 20q13.33 (rs6010620, *RTEL1*) (10, 13, 14). Telomerase reverse transcriptase (*TERT*) is crucial for telomerase activity in preserving telomeres and cellular immortality (15). In contrast, coiled-coil domain containing 26 (*CCDC26*) downregulates telomerase activity and increases apoptosis. Cyclin-dependent kinase inhibitor 2A/B (*CDKN2A/B)* can partially control the cell cycle. Regulator of telomere elongation helicase 1 (*RTEL1*), as a DNA helicase, can inhibit homologous recombination for directly maintaining the genomic stability (16). These genes all have varied degrees of influence on glioma pathogenesis. Due to limited sample sizes or relatively homogeneous ethnicities, current literature investigating the relationship between glioma and SNPs susceptibility frequently resulted in inconsistent findings.

In this meta-analysis, we expanded sample size by enrolling up 21 articles and used five genetic models to examine the relationship between glioma and SNPs of *TERT* (RS2736100), *CCDC26* (RS4295627), *CDKN2A/B* (RS497756), and *RTEL1* (rs6010620).

## 2 Materials and methods

The meta-analysis in the study was performed as per the Preferred Reporting Items for Systematic reviews and Meta-Analysis version.

### 2.1 Literature retrieval

Literature search was carried out comprehensively in eight databases, namely PubMed, Embase, WoS, Scopus, Cochrane Library, and Google Scholars. The MeSH term were as follows: “((Nucleotide Polymorphism, Single) OR (SNP) OR (Polymorphisms, Single Nucleotide) OR (SNP) OR (SNP) OR (SNPs) OR (variation) OR (mutation)) AND ((Gliomas) OR (Glioma) OR (Glial Cell Tumors) OR (Glial Cell Tumor) OR (Tumor, Glial Cell) OR (Tumors, Glial Cell) OR (Mixed Glioma) OR (Glioma, Mixed) OR (Malignant Glioma) OR (Glioma, Malignant) OR (Astrocytoma) OR (Glioblastoma) OR (Diffuse Intrinsic Pontine Glioma) OR (Ependymoma) OR (Glioma, Subependymal) OR (Ganglioglioma) OR (Medulloblastoma) OR (Oligodendroglioma) OR (Optic Nerve Glioma)) AND (rs xxxxxxx)”. In addition, we screened relevant reviews and references from previous meta-analyses to increase the number of other eligible studies. The retrieval process was performed by two researchers independently. Any discrepancy was adjudicated by a senior investigator.

### 2.2 Inclusion criteria

(1) case-control or cohort study and genome-related study regarding the association of rs2736100 polymorphism of *TERT* gene, rs4295627 polymorphism of *CCDC26* gene, rs4977756 polymorphism of *CDKN2A/B* gene and rs6010620 polymorphism of RTEL gene with glioma susceptibility;
(2) studies containing allelic and genotypic data or ORs and 95% CIs for susceptibility;
(3) appropriate statistical methods and reliable data with clear and unambiguous expression of results, allowing calculation of the ORs and 95% CIs;
(4) no overlapped data, and studies involving the same data or overlapped by the same authors, only the one that had the largest sample size or the most recently published.

### 2.3 Exclusion criteria

(1) review, commentary, abstract, and case report types;
(2) articles with original literature content did not involve *TERT* (rs2736100), *CCDC26* (rs4295627), *CDKN2A/B* (rs4977756) and *RTEL1* (rs6010620);
(3) articles were unrelated to glioma research;
(4) articles with insufficient data, such as those lacking gene frequency information or ORs with 95% CIs;
(5) articles with non-human study subjects.

### 2.4 Data extraction and literature quality evaluation

Each study covered the data of: the first author’s name, publication year, ethnicity (Caucasian, Asian, Multiple), number of cases and controls with relevant genotypes, ORs and 95% CIs. In addition, the genetic model used in the article was checked. We applied the Newcastle-Ottawa Scale for evaluating the literature quality. Two researchers performed independent data extraction and evaluated the literature quality. A senior investigator took charge of discrepancy arbitration.

### 2.5 Statistical analysis

STATA 17.0 MP was implemented for all statistical analyses in this study. First, we performed the Hardy-Weinberg Equilibrium (HWE) test (*p*<0.05 reported statistical significance). Second, we measured the association degree of *TERT* (rs2736100), *CCDC26* (rs4295627), *TERT* (rs2736100) and *RTEL1* (rs6010620) with glioma risk under five genetic models using the ORs and 95% CIs, which were the RM, O-DM, AM, C-DM and DM.

We merged the ORs and 95% CIs that satisfied the criteria under the same models. In addition, in all genetic models, subgroup analyses were carried out according to ethnic differences and the *I^2^* values were adopted for evaluating study heterogeneity. When *I^2^ ≤* 50%, a fixed effects model served for data analysis; when *I^2 =^* 50%-75%, a random effects model served for data analysis; and when *I^2^* >75%, the Galbraith plot method was used to attenuate the heterogeneity of the study. In order to statistically analyze publication bias, we applied the Egger’s Test (*p*<0.05: statistical significance).

## 3 Result

### 3.1 Literature retrieval and quality evaluation

**Figure 1:**
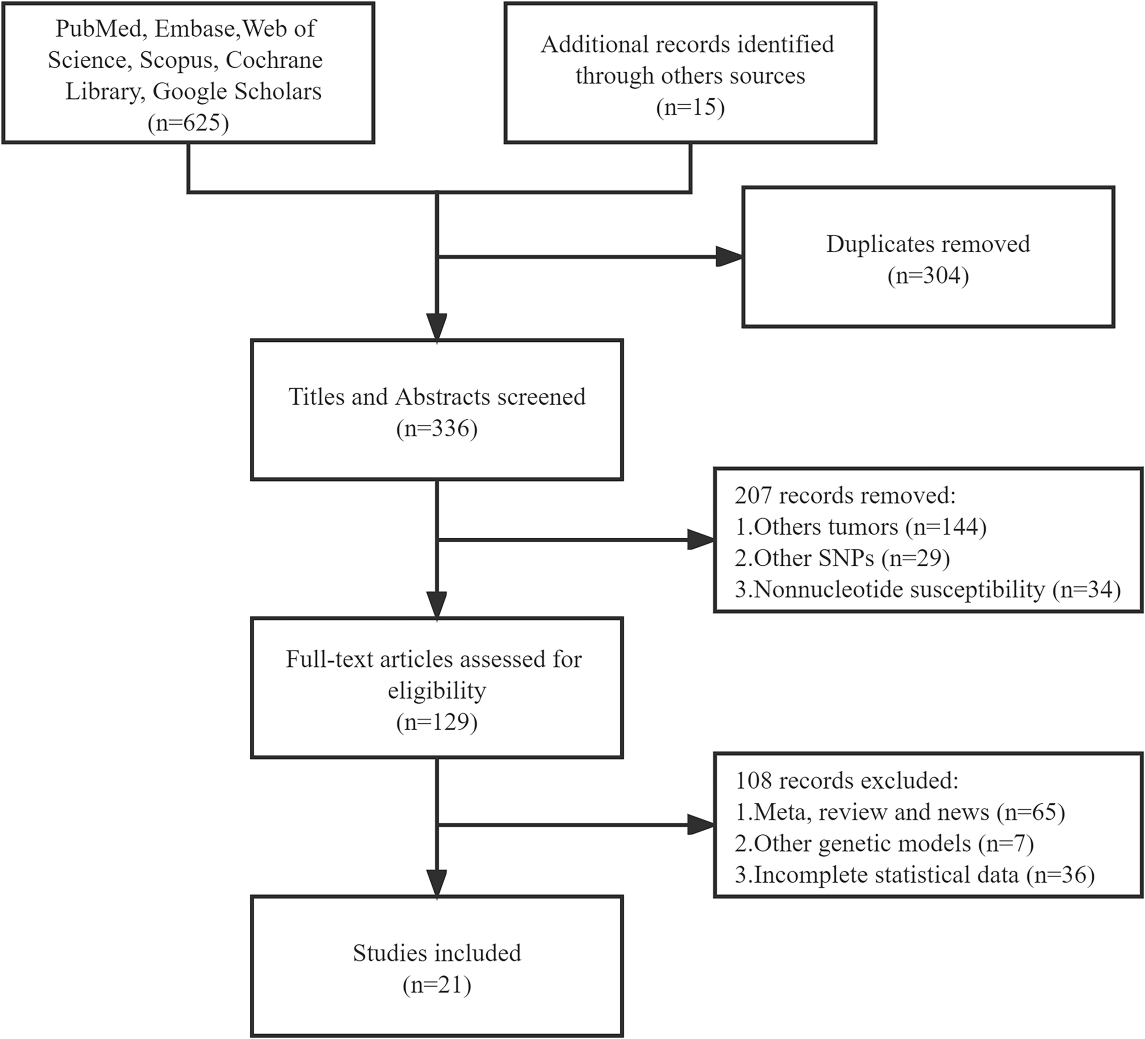
Flow chart of the study selection and exclusion criteria from this meta-analysis.

We initially searched 640 literature and eventually enrolled 21 eligible articles covering Caucasian, Asian, and others (10, 17–36). Twenty-one articles covered Caucasian, Asian, and multiracial studies. 15 out of the 21 studies were able to extract the complete genotype frequencies regarding the two groups, and we performed the HWE test on the control group (**Table 1**). Six studies were able to extract only ORs and 95% CIs under the correlation genetics model, as shown in **Table 2**. **Figure 1** displays the entire search process. The included articles have a moderate quality (**Table 3**).

**Table 1:** Main characteristics of the studies that provided genotype frequencies and were included in the meta-analysis.

SNP	Author	Year	Ethnicity	Case			Control			P (HWE)
				TT	TG	GG	TT	TG	GG	
rs2736100	Shete(French)	2009	Caucasian	225	686	441	383	807	371	balanced
	Shete(German)	2009	Caucasian	91	240	160	133	269	163	balanced
	Shete(Sweden)	2009	Caucasian	120	326	177	212	367	185	balanced
	Shete(UK)	2009	Caucasian	115	316	200	349	676	409	unbalanced
	Shete(USA)	2009	Caucasian	230	645	372	546	1103	584	balanced
	Chen	2011	Asian	244	515	194	334	542	160	unbalanced
	Pandith	2020	Caucasian	32	32	42	122	52	36	unbalanced
	Wrensch	2009	Multiple	95	354	242	1021	1904	1056	unbalanced
	Safaeian	2013	Multiple	164	402	244	789	1511	780	balanced
	Wang	2011	Caucasian	91	172	69	217	401	197	balanced
	Schoemaker(Demark)	2010	Caucasian	22	58	39	31	74	41	balanced
	Schoemaker(Finland)	2010	Caucasian	8	56	33	23	53	19	balanced
	Schoemaker(Sweden)	2010	Caucasian	29	107	57	101	171	90	balanced
	Schoemaker(UK-North)	2010	Caucasian	59	198	118	143	317	175	balanced
	Schoemaker(UK-South)	2010	Caucasian	53	105	74	86	202	107	balanced
rs4295627	Wang	2011	Caucasian	187	121	24	556	242	19	balanced
	Safaeian	2013	Caucasian	483	284	43	2025	935	123	balanced
	Shanqu	2012	Asina	121	92	12	127	102	25	balanced
	Di Stefano	2013	Caucasian	532	278	45	877	286	27	balanced
	Carl Wibom	2015	Caucasian	350	174	36	391	163	6	unbalanced
	Viana-Pereira	2020	Caucasian	79	35	0	128	42	4	balanced
	Shete(French)	2009	Caucasian	885	418	71	1133	421	25	unbalanced
	Shete(German)	2009	Caucasian	283	185	30	414	144	13	balanced
	Shete(Sweden)	2009	Caucasian	393	223	27	492	247	36	balanced
	Shete(UK)	2009	Caucasian	386	216	29	976	410	48	balanced
	Shete(USA)	2009	Caucasian	735	451	60	1496	667	72	balanced
	Schoemaker(Demark)	2010	Caucasian	76	40	7	98	46	3	balanced
	Schoemaker(Finland)	2010	Caucasian	47	34	16	58	31	6	balanced
	Schoemaker(Sweden)	2010	Caucasian	130	63	6	241	117	14	balanced
	Schoemaker(UK-North)	2010	Caucasian	237	122	16	434	156	27	balanced
	Schoemaker(UK-South)	2010	Caucasian	137	83	12	266	119	11	balanced
rs4977756	Wang	2011	Caucasian	113	151	68	303	389	125	balanced
	Di Stefano	2013	Caucasian	303	409	137	516	531	143	balanced
	Safaeian	2013	Multiple	248	383	179	1112	1460	509	balanced
	Fahmideh	2015	Caucasian	29	73	29	164	223	90	balanced
	Li	2012	Asian	139	72	15	152	84	15	balanced
	Sibin	2016	Asian	73	48	7	79	50	11	balanced
	Viana-Pereira	2020	Caucasian	34	58	26	82	72	22	balanced
	Shete(French)	2009	Caucasian	474	639	239	651	723	209	balanced
	Shete(German)	2009	Caucasian	151	240	108	211	265	90	balanced
	Shete(Sweden)	2009	Caucasian	150	325	157	223	379	168	balanced
	Shete(UK)	2009	Caucasian	189	604	138	501	662	270	balanced
	Shete(USA)	2009	Caucasian	377	594	276	782	1083	370	balanced
	Schoemaker(Demark)	2010	Caucasian	37	55	29	54	64	27	balanced
	Schoemaker(Finland)	2010	Caucasian	26	51	18	36	38	22	balanced
	Schoemaker(Sweden)	2010	Caucasian	42	97	57	110	177	80	balanced
	Schoemaker(UK-North)	2010	Caucasian	108	182	85	216	301	110	balanced
	Schoemaker(UK-South)	2010	Caucasian	70	113	70	141	181	68	balanced
rs6010620	Stefano	2013	Caucasian	21	234	582	48	377	765	balanced
	Wang	2011	Caucasian	15	99	218	49	296	472	balanced
	Safaeian	2013	Caucasian	26	245	539	148	1014	1921	balanced
	Fahmideh	2015	Caucasian	6	38	88	35	168	272	balanced
	Chen	2011	Asian	411	454	93	547	438	55	unbalanced
	Viana-Pereira	2020	Caucasian	2	33	86	10	56	113	balanced
	Wrensch	2009	Caucasian	44	409	978	213	1383	2395	balanced
	Jin	2013	Asian	204	181	48	236	202	24	unbalanced
	Li	2013	Asian	293	261	75	337	267	40	balanced
	Shete(French)	2009	Caucasian	34	386	912	59	508	978	balanced
	Shete(German)	2009	Caucasian	16	147	336	28	177	352	balanced
	Shete(Sweden)	2009	Caucasian	20	195	430	54	264	456	balanced
	Shete(UK)	2009	Caucasian	26	179	426	82	533	818	balanced
	Shete(USA)	2009	Caucasian	46	405	796	123	785	1327	balanced
	Schoemaker(Demark)	2010	Caucasian	1	38	83	8	56	83	balanced
	Schoemaker(Finland)	2010	Caucasian	4	22	69	3	30	63	balanced
	Schoemaker(Sweden)	2010	Caucasian	4	52	144	25	116	230	balanced
	Schoemaker(UK-North)	2010	Caucasian	18	106	252	44	212	376	balanced
	Schoemaker(UK-South)	2010	Caucasian	8	65	159	28	129	233	balanced

**Table 2:** Characteristics of the literature providing ORs and 95% CIs under the correlated genetic model.

SNP	Author	Year	Ethnicity	Contrast models	Odds ratio		
					ORs	[95% CIs]	
rs2736100	Rajaraman	2012	Caucasian	G allele vs. T allele (Allele model)	1.295	1.188	1.412
	Egan	2011	Caucasian		1.37	1.18	1.61
	Melin	2013	Caucasian		1.41	1.05	1.89
	Egan	2011	Caucasian	GG vs. TT (Co-dominance model)	1.96	1.41	2.70
	Melin	2013	Caucasian		1.87	1.06	3.30
	Egan	2011	Caucasian	GT vs. TT (Co-dominance model)	1.25	0.97	1.61
	Melin	2013	Caucasian		1.10	0.64	1.88
rs4295627	Rajaraman	2012	Caucasian	G allele vs. T allele (Allele model)	1.137	1.022	1.265
	Egan	2011	Caucasian		1.18	0.96	1.45
	Melin	2013	Caucasian		1.23	0.87	1.72
	Wei	2014	Asian		0.95	0.64	1.43
	Chen	2011	Asian		0.94	0.82	1.09
	Egan	2011	Caucasian	GG vs. TT (Co-dominance model)	1.05	0.55	2.00
	Melin	2013	Caucasian		1.58	0.67	3.71
	Egan	2011	Caucasian	GT vs. TT (Co-dominance model)	1.27	1.00	1.63
	Melin	2013	Caucasian		1.20	0.78	1.85
rs4977756	Rajaraman	2012	Caucasian	G allele vs. A allele (Allele model)	1.283	1.177	1.398
	Egan	2011	Caucasian		1.16	0.98	1.37
	Melin	2013	Caucasian		1.40	1.04	1.88
	Chen	2011	Asian		1.06	0.91	1.23
	Egan	2011	Caucasian	GG vs. AA (Co-dominance model)	1.29	0.92	1.80
	Melin	2013	Caucasian		2.10	1.11	3.95
	Egan	2011	Caucasian	GA vs. AA (Co-dominance model)	1.32	1.02	1.71
	Melin	2013	Caucasian		2.01	1.15	3.52
rs6010620	Rajaraman	2012	Caucasian	G allele vs. A allele (Allele model)	1.427	1.280	1.590
	Egan	2011	Caucasian		1.37	1.12	1.67
	Melin	2013	Caucasian		2.564	1.639	4
	Egan	2011	Caucasian	GG vs. AA (Co-dominance model)	1.92	1.09	3.45
	Melin	2013	Caucasian		0.09	0.01	0.66
	Yang	2015	Asian		8.22	3.11	21.72
	Egan	2011	Caucasian	GA vs. AA (Co-dominance model)	1.37	1.06	1.75
	Melin	2013	Caucasian		0.43	0.26	0.70
	Yang	2015	Asian		1.13	0.64	2.01
	Yang	2015	Asian	GG+AA vs.GA (Overdominance model)	1.220	0.709	2.083
	Yang	2015	Asian	GG+AG vs.AA (Dominance model)	1.54	0.90	2.63

**Table 3:** Results of literature quality evaluation.

No.	Study	Year	Selection				Comparability control for important factor	Exposure			Scores
			Adequate definition of cases	Representativeness of the cases	Selection of controls	Definition of controls		Ascertainment of exposure	Same method of ascertainment for case and controls	Non-responserate	
1	Wang	2011	*	*		*	**	*	*	*	8
2	Safaeian	2013	*	*	*	*	**	*			7
3	Li	2012	*	*		*	*	*	*	*	7
4	Stefano	2013	*	*		*	*		*		5
5	Wibom	2015	*	*		*	**		*	*	7
6	Viana-Pereira	2020	*	*		*	*		*	*	6
7	Shete	2009	*	*		*	**	*	*	*	8
8	Schoemaker	2010	*	*	*	*	**		*		7
9	Fahmideh	2015		*	*	*	*		*		5
10	Sibin	2016	*	*	*	*			*		5
11	Chen	2011	*	*		*		*	*	*	6
12	Wrensch	2009	*	*	*	*	**		*		7
13	Jin	2013	*	*		*		*	*		5
14	Li	2013	*	*		*	**	*	*		7
15	Pandith	2020	*	*		*	*		*		5
16	Rajaraman	2012		*		*	**	*	*		6
17	Egan	2011		*	*	*	*		*		5
18	Melin	2013	*	*		*	**	*	*		7
19	Wei	2014	*	*	*	*		*	*		6
20	Yang	2015	*	*		*	*	*	*		6
21	Melin	2017	*	*	*	*	**	*	*	*	9

### 3.2 Meta-analysis results

#### 3.2.1 rs2736100

**Figure 2:**
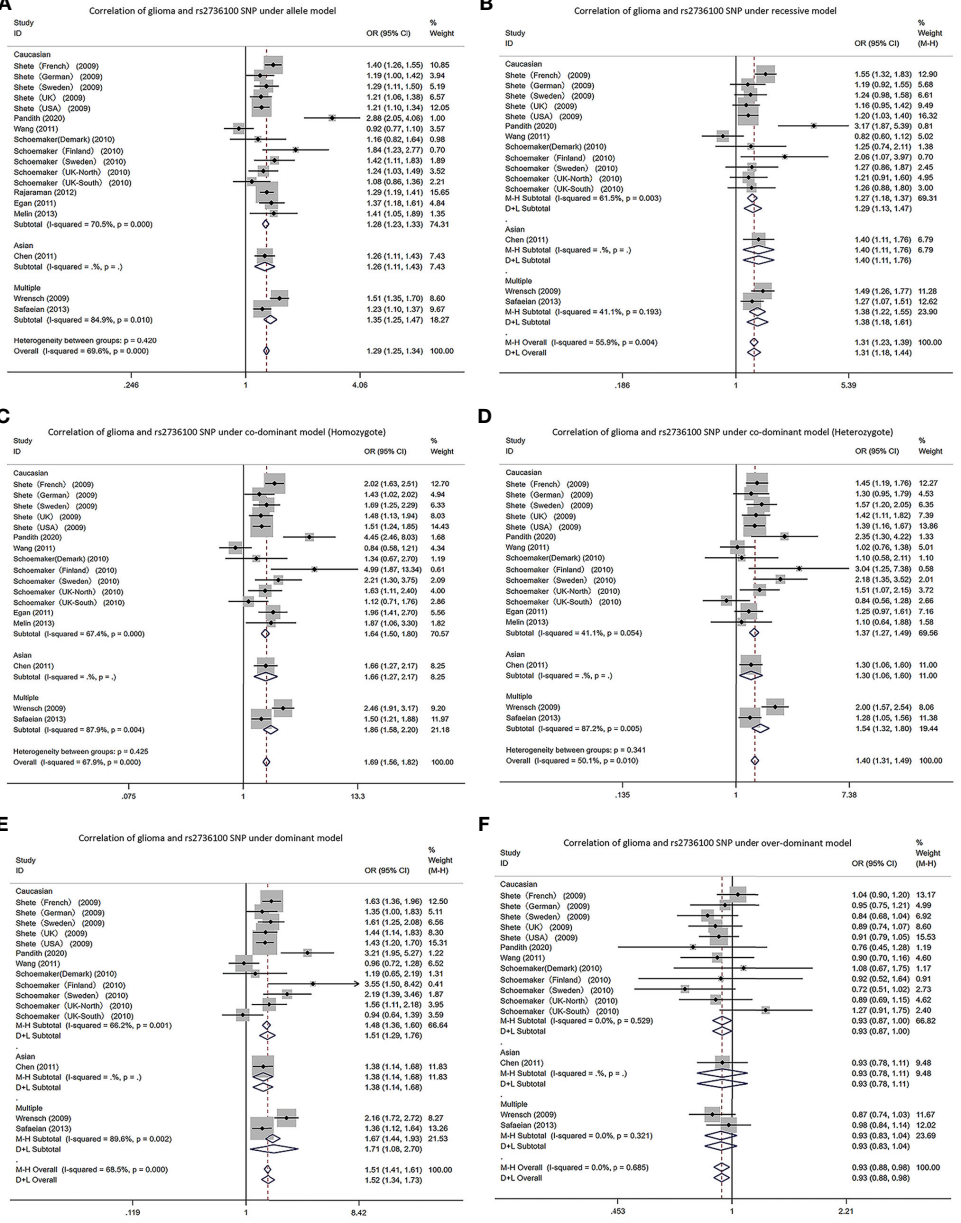
Forest plots of meta-analyses for correlation of glioma and rs2736100 SNP under all models. **(A)** allele model; **(B)** recessive model; **(C)** co-dominant model (Homozygote); **(D)** co-dominant model (Heterozygote); **(E)** dominant model; **(F)** over-dominant model.

In the *TERT* gene rs2736100 polymorphism, allele G is the risk gene (**Figure 2**). According to the meta-analysis analysis, the *TERT* gene rs2736100 polymorphism led to increased glioma risk in 5 genetic models (AM (G vs T): OR=1.29, 95% CI: 1.25 to 1.34; RM (GG vs TT+TG): OR=1.31, 95% CI: 1.18 to 1.44; DM (TG+GG vs TT): OR=1.52, 95% CI: 1.34 to 1.73; C-DM (GG vs TT): OR=1.69, 95% CI: 1.56 to 1.82; C-DM (GT vs TT): OR=1.40, 95% CI: 1.31 to 1.49; O-DM (TT+GG vs TG): OR=0.93, 95% CI: 0.88 to 0.98). When stratifying the analysis according to ethnicity, we obtained similar results in Caucasians, although in the O-DM results only showed a trend of association with glioma (AM (G vs T): OR=1.28, 95% CI: 1.23 to 1.33; RM (GG vs TT+TG): OR=1.29, 95%CI: 1.13 to 1.47; DM (TG+GG vs TT): OR=1.51, 95% CI: 1.29 to 1.76; C-DM (GG vs TT): OR=1.64, 95% CI: 1.50 to 1.80; C-DM (GT vs TT): OR=1.37, 95% CI: 1.27 to 1.49; O-DM (TT+GG vs TG): OR=0.93, 95% CI: 0.87 to 1.00). Among Asians, we included only one study, and the results were similar to those of Caucasians. In the study of a multi-ethnic population, we included the study of Wrensch and Safaeian which revealed the relevance of the *TERT* gene rs2736100 polymorphism to increased glioma risk.

#### 3.2.2 rs4295627

**Figure 3:**
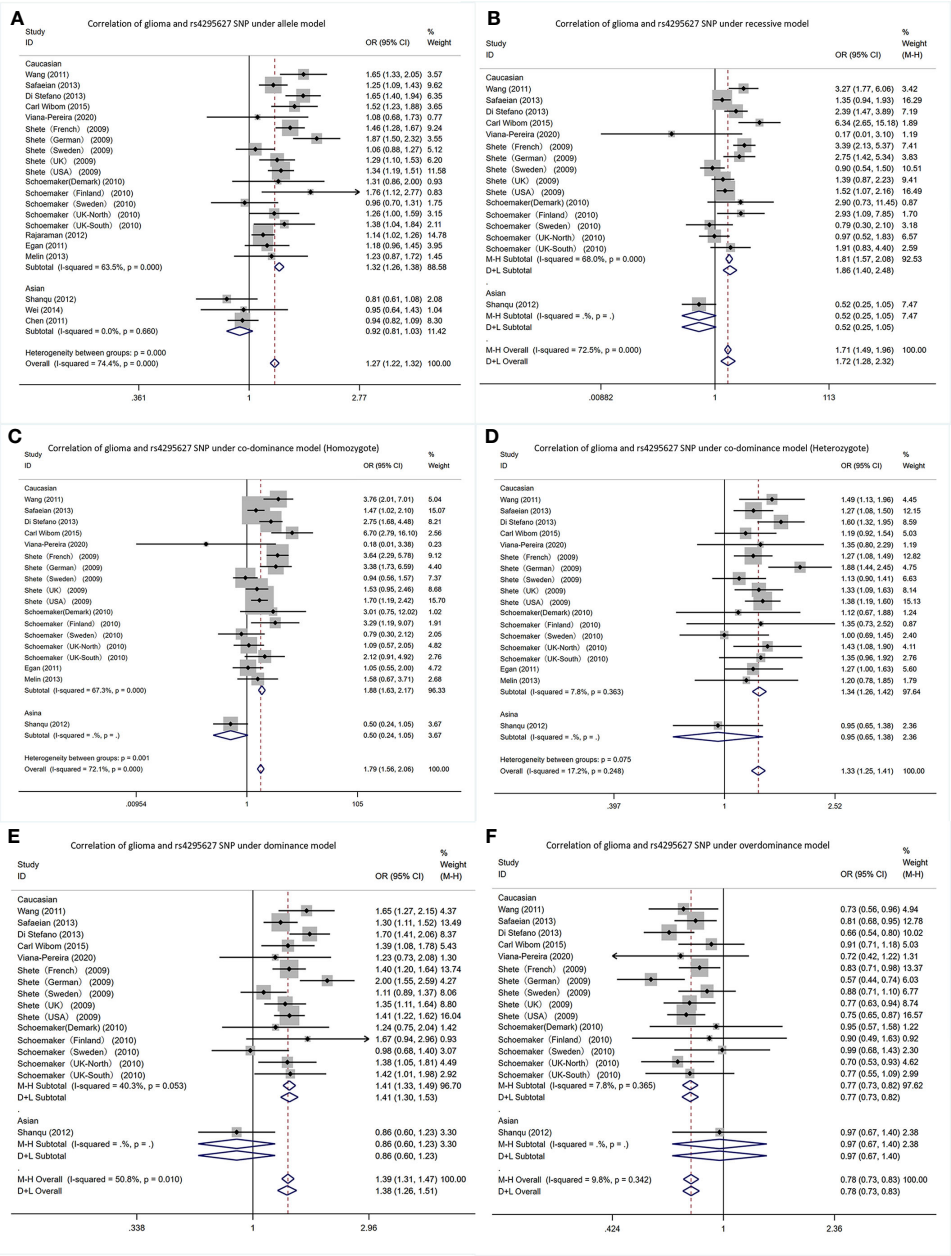
Forest plots of meta-analyses for correlation of glioma and rs4295627 SNP under all models. **(A)** allele model; **(B)** recessive model; **(C)** co-dominant model (Homozygote); **(D)** co-dominant model (Heterozygote); **(E)** dominant model; **(F)**. over-dominant model.

In the *CCDC26* gene rs4295627 polymorphism, allele T was the risk gene (**Figure 3**). Among Caucasians, according to the meta-analysis results: AM (G vs T): OR= 1.32; 95% CI: 1.26 to 1.38; RM (GG vs TT+TG): 1.86; 1.40 to 2.48; DM (TG+GG vs TT): 1.41; 1.33 to 1.49; C-DM (GG vs TT): 1.88; 1.63 to 2.17; C-DM (GT vs TT): 1.34; 1.26 to 1.42; O-DM (TT+GG vs TG): 0.77; 0.73 to 0.82.

In Asians, in the Shanqu, Wei and Chen AM (G vs T): OR=0.92, 95% CI: 0.81 to 1.03. In one study, the RM (GG vs TT+TG): 0.52; 0.25~1.05; DM (TG+GG vs TT): 0.86; 0.60 to 1.23; C-DM (GG vs TT): 0.50; 0.24 to 1.05; C-DM (GT vs TT): 0.95; 0.65 to 1.38; O-DM (TT + GG vs TG): 0.97; 0.67 to 1.40.

#### 3.2.3 rs4977745

**Figure 4:**
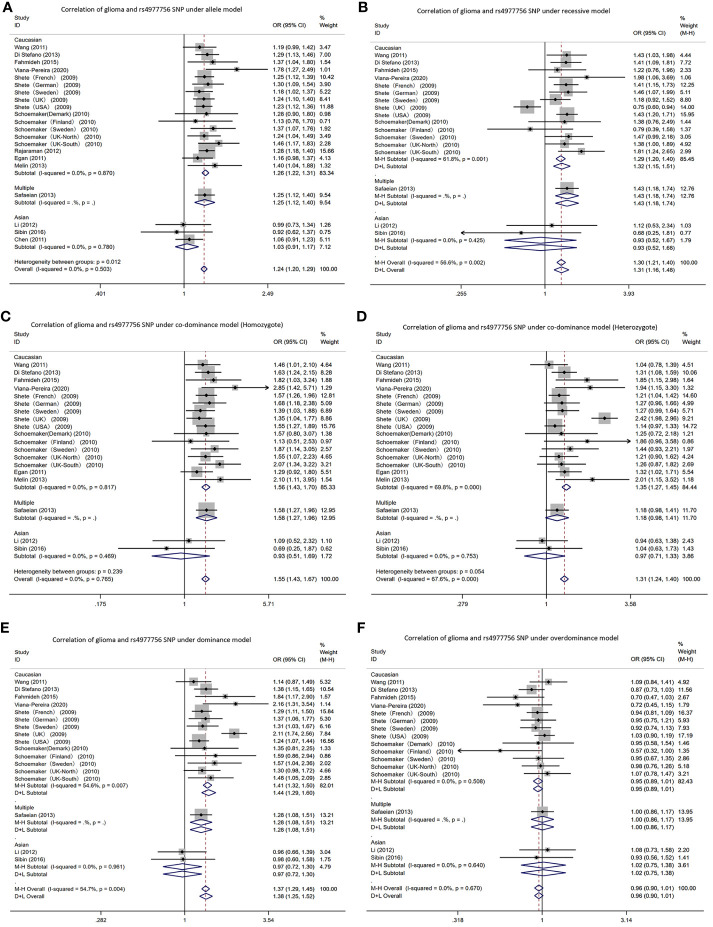
Forest plots of meta-analyses for correlation of glioma and rs4977756 SNP under all models. **(A)** allele model; **(B)** recessive model; **(C)** co-dominant model (Homozygote); **(D)** co-dominant model (Heterozygote); **(E)** dominant model; **(F)** over-dominant model.

In the *CDKN2A/B* gene rs4977756 polymorphism, allele T was the risk gene (**Figure 4**). Among Caucasians, according to the meta-analysis results: AM (G vs A): OR=1.26, 95% CI: 1.22 to 1.31; RM (GG vs AA+AG): 1.32; 1.15 to 1.51; DM (AG+GG vs AA): 1.44; 1.29 to 1.60; C-DM (GG vs AA): 1.56; 1.43 to 1.70; C-DM (GA vs AA): 1.35; 1.27 to 1.45; O-DM (AA+GG vs AG): 0.95; 0.89 to 1.01.

In Asians, in the Li, Sibin and Chen AM (G vs A): OR=1.03, 95% CI: 0.91 to 1.17. In one study, the RM (GG vs AA+AG): 1.12; 0.53 to 2.34; DM (AG+GG vs AA): 0.96; 0.66 to 1.39; C-DM (GG vs AA): 1.09; 0.52 to 2.32; C-DM (GA vs AA): 0.94; 0.63 to 1.38; O-DM (AA+GG vs AG): 1.08; 0.73 to 1.58. In other study, RM (GG vs AA+AG): 0.68; 0.25 to 1.81; DM (AG+GG vs AA): 0.98; 0.60 to 1.58; C-DM (GG vs AA): 0.69; 0.25 to 1.87; C-DM (GA vs AA): 1.04; 0.63 to 1.73; O-DM (AA+GG vs AG): 0.93; 0.56 to 1.52.

#### 3.2.4 rs6010620

**Figure 5:**
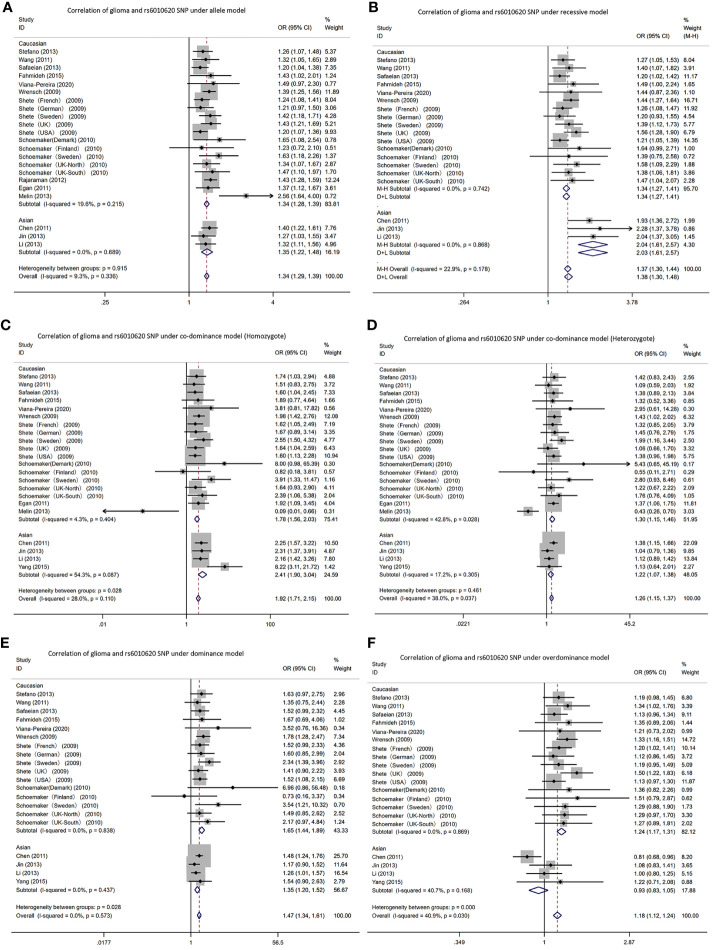
Forest plots of meta-analyses for correlation of glioma and rs6010620 SNP under all models. **(A)** allele model; **(B)** recessive model; **(C)** co-dominant model (Homozygote); **(D)** co-dominant model (Heterozygote); **(E)** dominant model; **(F)**. over-dominant model.

In the *RTEL1* gene rs6010620 polymorphism, allele T was the risk gene (**Figure 5**). Among Caucasians, according to the meta-analysis: AM (G vs A): OR=1.34, 95% CI: 1.28 to 1.39; RM (GG vs AA+AG): 1.34; 1.27 to 1.41; DM (AG+GG vs AA): 1.65; 1.44 to 1.89; C-DM (GG vs AA): 1.78; 1.56 to 2.03; C-DM (GA vs AA): 1.30; 1.15 to 1.46; O-DM (AA+GG vs AG): 1.24; 1.17 to 1.31.

In Asians, based on the meta-analysis results: AM (G vs A): OR=1.35, 95% CI: 1.22 to 1.48; RM (GG vs AA+AG): 2.04; 1.61 to 2.57; DM (AG+GG vs AA): 1.35; 1.20 to 1.52; C-DM (GG vs AA): 2.41; 1.09 to 3.04; C-DM (GA vs AA): 1.22; 1.07 to 1.38; O-DM (AA+GG vs AG): 0.93; 0.83 to 1.05.

### 3.3 Publication bias

Our study adopted Egger’s Test for evaluating the literature publication bias and the results are shown in **Table 4**. P > 0.05 indicates that publication bias did not exist.

**Table 4:** Publication bias results of glioma SNPs under five genetic models.

SNP	Allele model	Recessive model	Dominance model	Co-dominance model (Homozygote)	Co-dominance model (Heterozygote)	Overdominance model
rs2736100	0.469	0.666	0.581	0.530	0.622	0.663
rs4295627	0.949	0.926	0.426	0.924	0.436	0.427
rs4977756	0.984	0.812	0.633	0.893	0.511	0.111
rs6010620	0.081	0.010	0.014	0.674	0.604	0.461

In rs2376100, there was publication bias in AM (p=0.469). In rs4295627, publication bias existed in dominant, co-dominant and O-DM (DM (TG+GG vs TT): p=0.426; C-DM (GT vs TT): p=0.436; O-DM (TT+GG vs TG): p=0.427). In rs4977756, publication bias existed in O-DM (p=0.111). In rs6010620, publication bias existed in the recessive, dominant and O-DM (RM (GG vs AA+AG): p=0.010; DM (AG+GG vs AA): p=0.014; O-DM (AA+GG vs AG): p= 0.461).

## 4 Discussion

Our study is one of the first ones with conducting the first meta-analysis with the largest sample size as far as we concern and we performed detailed analyses of multiple associated SNPs under five genetic models. Besides, stratified analyses were carried out considering ethnicity. This indicates reliable meta-analysis results.

From the meta-analysis, allele G in the SNP at the rs2736100 locus differentially elevated glioma risk, which conforms to studies by Di Stefano (24). In particular, in the C-DM (GG vs TT), it elevated the risk by 69% (95% CI: 1.56 to 1.82). However, in the O-DM (TT+GG vs TG), the risk of glioma was reduced by 7% (95% CI: 0.88 to 0.98). We obtained basically identical results in Caucasians after stratification by ethnicity. Nevertheless, in the Asian O-DM, the risk of glioma was not associated with the SNP of the gene. Nevertheless, there is only one study on Asian populations and it is necessary to cautiously interpret the obtained results. Caucasians and Asians showed significant differences in the SNPs at the rs4295627 locus versus the rs4977756 locus. In the allele, recessive, dominant and C-DMs, all increased the risk of glioma. But in Asian populations, SNPs at both loci did not present relevance to glioma risk. This suggests the existence of significant racial differences. However, the amount of research literature on Asian populations is too small and further studies are needed to verify this conclusion. Among the SNPs at the rs6010620 locus, according to analysis of all five models, allele G elevated the risk, with the C-DM (GG vs AA) increasing the risk by 92% (95% CI: 1.71 to 2.15). However, in Chen’s study, the risk of glioma was reduced by 19% (95% CI: 0.68 to 0.96) in the O-DM, which was possibly because the sample size in Chen’s study was small (19).

Telomere refers to a small fragment of DNA-protein complex located at the end of eukaryotic chromosome, consisting of a six-base repeat sequence -TTAGGG- and binding proteins, which have important roles in localizing, replicating and protecting chromosome and controlling cell growth, and exert a close relevance to apoptosis, transformation and immortalization of cells. Telomere shortening may also increase susceptibility to cancer (37). *TERT* is the most important component in regulating telomerase activity. A few scholars found the relevance of *TERT* expression to glioma grade and patients’ prognosis (38). Located in intron 2 in *TERT*, the SNP can impact the telomerase activity or clearly relates to a functional variant in *TERT* (39). In the study by Codd, people who possessed TT genotype (h*TERT* rs2736100 T > G) presented shorter telomeres relative to people who possessed TG genotype (40). Oppositely, a larger number of cell divisions may be supported at long telomeres, which makes it easier to achieve abnormalities and thereby contributes to the development of cancers (41). However, in our study, in the SNP of *TERT* rs2736100, allele G increases the risk of developing glioma. This implicates that telomere length possibly plays a double-edged role in cancer development. In 2019, Yuan conducted a detailed review of *TERT* mutation-related cancers, with the highest frequency of *TERT* mutation reaching 80% in glioma, and more than a dozen tumors such as hepatocellular carcinoma, thyroid cancer, and skin melanoma are also closely related to this gene mutation (42). Hence, it is necessary to conduct deep investigations on relevant function mechanisms in order to better treat clinical patients.

*CCDC26* can be found on chromosome 8q24.21, and the rs4295627 single nucleotide polymorphism can only be found in the intron 3 region regarding the *CCDC26* gene, where a G-T base mutation can increase glioma disease risk (31). This is consistent with the meta-analysis in the study. The explanation can be attributed to that retinoic acid phosphorylates cAMP response element binding proteins for the induction of caspase 8 transcription and downregulates the telomerase activity for enhancing the apoptosis in neuroblastoma and glioblastoma cells under death stimuli (43). In addition, clinical studies employ *CCDC26* to predict glioma patients’ diagnosis and prognosis (22). Jenkins found that *CCDC26* variants could remarkably elevate the risk of low-grade gliomas that underwent IDH1 or IDH2 mutations, suggesting the interaction between *CCDC26* and IDH mutations and/or the downstream effects regarding above mutations for promoting glioma development (44). Wang studies showed that lncRNA *CCDC26* silencing inhibits glioma cell growth and migration by targeting miR-203. It revealed the regulatory mechanism of *CCDC26*/miR-203 pathway in glioma pathogenesis and provided a new target for glioma treatment (45). In addition to gliomas, Homer-Bouthiette found that deletion of the gene reduced breast cancer incidence in a mouse model of breast cancer (46). This chromosomal region has also been studied in prostate, bladder and rectal cancers (47–50).

*CDKN2A/B* is a representative altered genes in human cancers, and expression deletion facilitates malignant behavior by dysregulating cell cycle and promoting cell proliferation (51). *CDKN2A/B* is localized at 9p21, encoding p14, p16 (CDKN2A) and p15 (CDKN2B) oncoproteins which play multiple roles in cellular stress recognition, senescence regulation, differentiation as well as apoptosis in the developmental and proliferative phases of cells (51, 52). As reported, an unknown antisense lncRNA is encoded at human *CDKN2A/B* locus at 9p21.3, i.e. ANRIL, which crucially impacts disease development. The rs4977756’s 59 kb telomere is also mapped to CDKN2B in the 122 kb region regarding LD 9p21.3. Based on the meta-analysis, the G allele elevates the glioma risk. It is suspected that this SNP alters the expression level of ANRIL and thus affects cell proliferation, apoptosis and metastasis to promote tumorigenesis (53). Interestingly, type 2 diabetes and cardiovascular disease also appear to be linked to this gene (54, 55).

*RTEL1* is involved in deoxyribonucleic acid repair, regulation of replication and transcription, and maintenance of telomere length. *RTEL1* can both promote and suppress tumorigenesis. *RTEL1* SNPs associated with gliomas are mainly found in the non-coding (intron) regions of genes. Intron SNPs possibly facilitate aberrant exon inclusion or deletion by altering mRNA splicing and the formation and addition regarding nonsense transcripts (56, 57). The *RTEL1* gene rs6010620 on 20q13.33 is a vital candidate genetic variant that has been widely reported (58). As reported by previous studies, mutations in the rs6010620 gene may cause gliomas, but these findings have not been confirmed. According to meta-analysis, in the five genetic models, rs6010620 increased glioma susceptibility, most significantly in the C-DM (GG vs AA), which increased the susceptibility by 92% (OR: 1.92; CI (1.71, 2.15). Also, miR-4530 underwent downregulation in glioma tissues as well as cell lines, and overexpression inhibited malignant biological behaviors like glioma cell migration, proliferation, invasion and colony formation. *RTEL1* was a direct target of miR-4530. The abnormal expression of *RTEL1* could lead to obvious reversion of the miR-4530 overexpression in glioma cell lines. The miR-4530/*RTEL1* axis acts as a latent treating target specific to gliomas (59). Not only glioma, but also mutated *RTEL1* has been linked to the development of astrocytoma and Hoyeraal-Hreidarsson syndrome (60). Although recent in-depth studies have confirmed that *RTEL1* can maintain genomic stability by studying its ability to maintain telomere homeostasis and promote DNA replication, there are still a lot of questions that shall be highlighted. Nowadays, researches have not confirmed the regulation of *RTEL1* and its recruitment to impact the replication forks and telomeres (60).

Studying the association between the risk of glioma and SNPs is definitive, because it could help clinicians use such polymorphisms as pragmatic molecular biomarkers of glioma risk, assist drug developers innovate relevant targeted therapeutic agents for the benefit of patients, and allow for basic researchers to explore the molecular mechanisms of glioma pathogenesis and accurately regulate glioma progression. According to meta-analysis results, racial differences exist in the risk of glioma, but there is a paucity of studies on Asian ethnicity, which requires researchers to conduct relevant studies. Exploration of SNPs at other loci and risk of glioma is also necessary.

## 5 Limitations

Some limitations should be noted when interpreting the current results. The study also presents some limitations, which shall be noted during the interpretation of current studies.

First, there were scarce studies on Asian populations. Studies on results in Asian populations should be interpreted with caution.

In addition, the absence of more specific individual information about interaction within genes and that between gene and environment prohibited us from conducting a more precise analysis.

Finally, some unavoidable publication bias in the results of meta-analysis should also be taken into account.

## 6 Conclusion

In summary, our study suggested that the *TERT* gene rs2736100 polymorphism, *CCDC26* gene rs4295627 polymorphism, *CDKN2A/B* gene rs4977756 polymorphism and *RTEL1* gene rs6010620 polymorphism might all increase the risk of glioma development, but there are ethnic differences. It is necessary to well develop case-control studies with large sample sizes and a focus on more races or glioma types to overcome the limitations described earlier to make the conclusions more accurate.

## Author contributions

All authors contributed to the article and approved the submitted version. YQW: Writing articles, searching literature and extracting literature. JZho and ZT: Analyzing the quality of articles. JZha: Analyzing Data. XC: Providing methodology. SL: Editing of articles.
