# Erratum: Pertinence of glioma and single nucleotide polymorphism of TERT, CCDC26, CDKN2A/B, and RTEL1 genes in glioma: a meta-analysis

**DOI:** 10.3389/fonc.2024.1393063

**Published:** 2024-03-11

**Authors:** Frontiers Production Office

**Affiliations:** Frontiers Media SA, Lausanne, Switzerland

**Keywords:** glioma, single nucleotide polymorphism, risk, meta-analysis, genetic model

Due to a production error, there was a mistake in the name of **Figure 2** as published. The figure part labels read as ‘Correlation of glioma and rs6010620 SNP’. The correct text is ‘Correlation of glioma and rs2736100 SNP’. The corrected **Figure 2** appears below.

**Figure 2:**
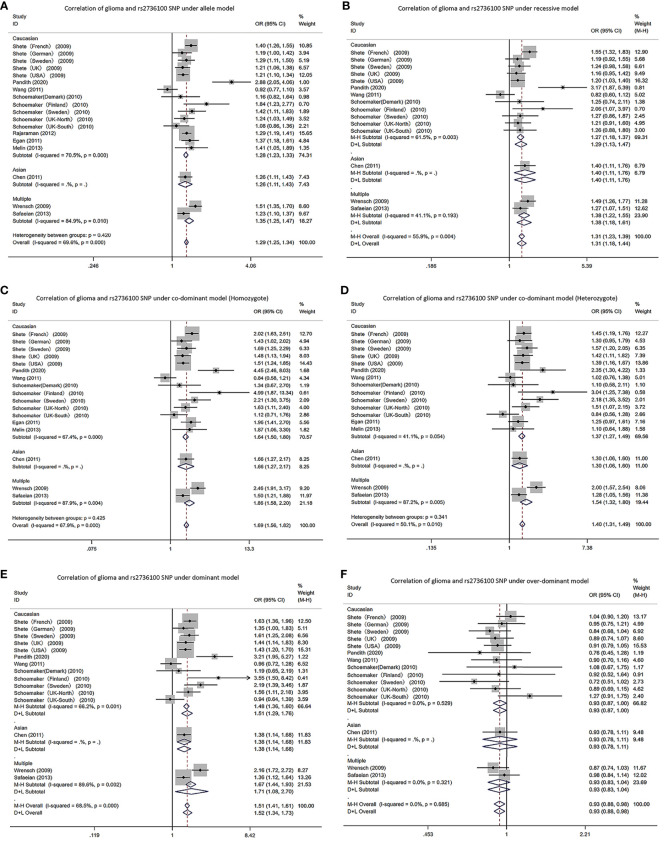
Forest plots of meta-analyses for correlation of glioma and rs2736100 SNP under all models. **(A)** allele model; **(B)** recessive model; **(C)** co-dominant model (Homozygote); **(D)** co-dominant model (Heterozygote); **(E)** dominant model; **(F)** over-dominant model.

Due to a production error, there was a mistake in the name of **Figure 4** as published. The figure part labels read as ‘Correlation of glioma and rs2736100 SNP’. The correct text is ‘Correlation of glioma and rs4977756 SNP.’ The corrected **Figure 4** appears below.

**Figure 4:**
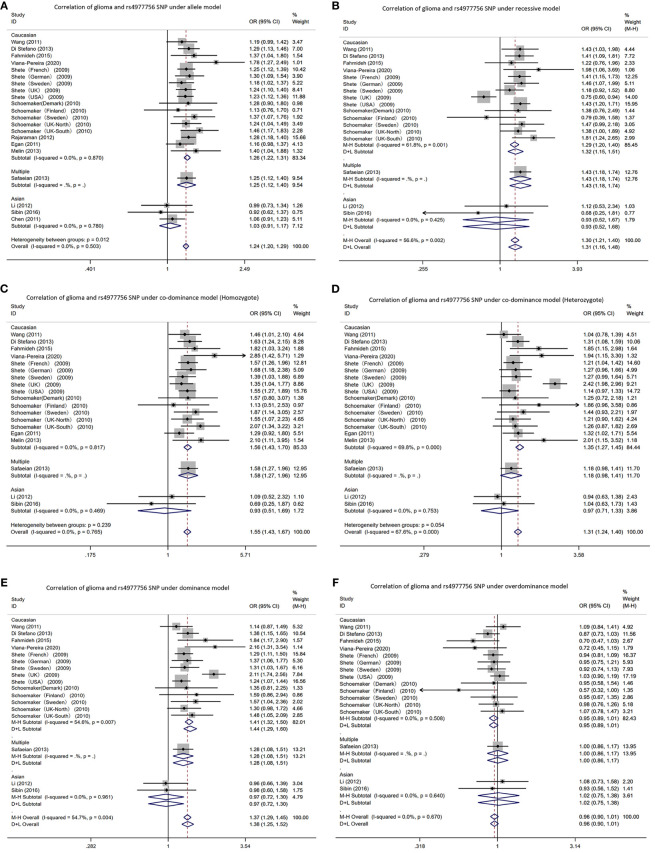
Forest plots of meta-analyses for correlation of glioma and rs4977756 SNP under all models. **(A)** allele model; **(B)** recessive model; **(C)** co-dominant model (Homozygote); **(D)** co-dominant model (Heterozygote); **(E)** dominant model; **(F)** over-dominant model.

Due to a production error, there was a mistake in the name of **Figure 5** as published. The figure part labels read as ‘Correlation of glioma and rs4977756 SNP’. The correct text is ‘Correlation of glioma and rs6010620 SNP.’ The corrected **Figure 5** appears below.

**Figure 5:**
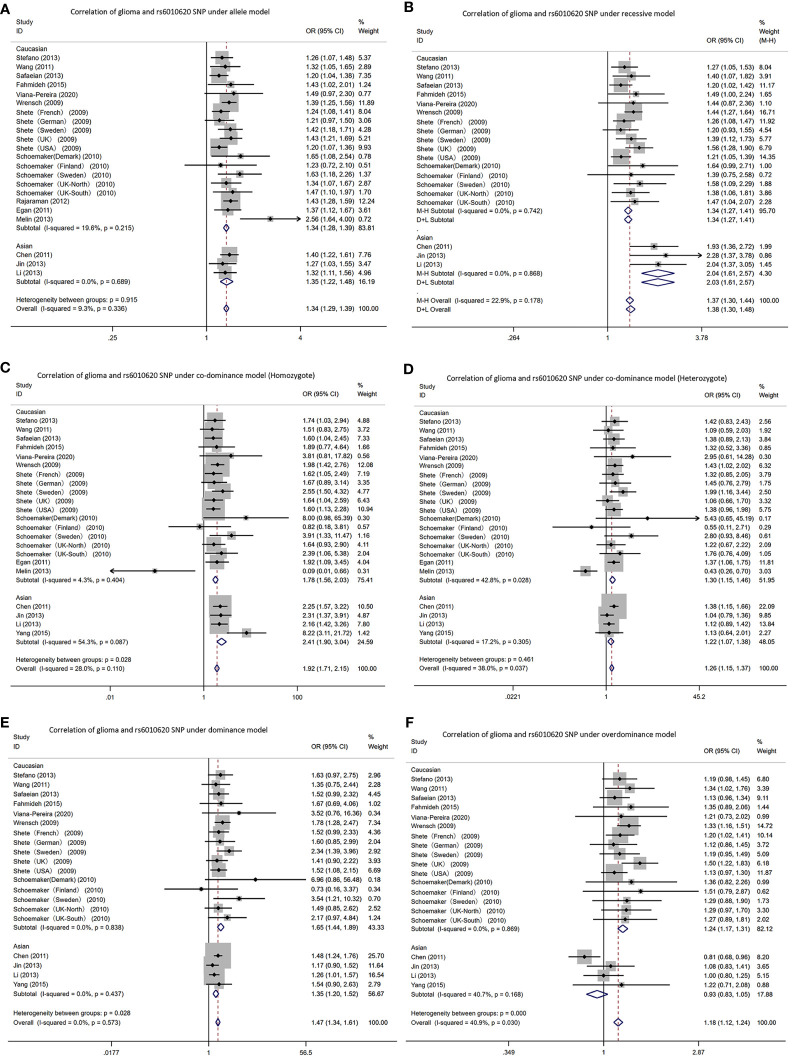
Forest plots of meta-analyses for correlation of glioma and rs6010620 SNP under all models. **(A)** allele model; **(B)** recessive model; **(C)** co-dominant model (Homozygote); **(D)** co-dominant model (Heterozygote); **(E)** dominant model; **(F)**. over-dominant model.

The publisher apologizes for this mistake.

The original version of this article has been updated.

